# *Fad104*, a Positive Regulator of Adipocyte Differentiation, Suppresses Invasion and Metastasis of Melanoma Cells by Inhibition of STAT3 Activity

**DOI:** 10.1371/journal.pone.0117197

**Published:** 2015-02-11

**Authors:** Daiki Katoh, Makoto Nishizuka, Shigehiro Osada, Masayoshi Imagawa

**Affiliations:** Department of Molecular Biology, Graduate School of Pharmaceutical Sciences, Nagoya City University, 3–1 Tanabe-dori, Mizuho-ku, Nagoya, Aichi, 467–8603, Japan; Michigan State University, UNITED STATES

## Abstract

Metastasis is the main cause of death in patients with cancer, and understanding the mechanisms of metastatic processes is essential for the development of cancer therapy. Although the role of several cell adhesion, migration or proliferation molecules in metastasis is established, a novel target for cancer therapy remains to be discovered. Previously, we reported that *fad104* (factor for adipocyte differentiation 104), a regulatory factor of adipogenesis, regulates cell adhesion and migration. In this report, we clarify the role of *fad104* in the invasion and metastasis of cancer cells. The expression level of *fad104* in highly metastatic melanoma A375SM cells was lower than that in poorly metastatic melanoma A375C6 cells. Reduction of *fad104* expression enhanced the migration and invasion of melanoma cells, while over-expression of FAD104 inhibited migration and invasion. In addition, melanoma cells stably expressing FAD104 showed a reduction in formation of lung colonization compared with control cells. FAD104 interacted with STAT3 and down-regulated the phosphorylation level of STAT3 in melanoma cells. These findings together demonstrate that *fad104* suppressed the invasion and metastasis of melanoma cells by inhibiting activation of the STAT3 signaling pathway. These findings will aid a comprehensive description of the mechanism that controls the invasion and metastasis of cancer cells.

## Introduction

Cancer is the second leading cause of death worldwide. Approximately 90% of all cancer deaths arise from the invasion and metastatic spread of primary tumors. Melanoma is one of the most lethal forms of skin cancer. Although it is a relatively rare cancer, its incidence has increased rapidly in the last few decades [1, 2]. If diagnosed early, melanoma is well curable by surgical resection. However, the lethality of melanoma is high because of its high metastatic potential. Therefore, clarification of the mechanisms by which the invasion and metastasis of melanoma cells are regulated is essential for the development of more effective therapies. Although it is known that the factors that regulate cell adhesion and migration are involved in the invasion and metastasis of melanoma cells, little is known about the molecular mechanism of invasion and metastasis.

Previously, to elucidate the mechanism of adipocyte differentiation, we isolated several novel genes whose expression was up-regulated in the early stage of adipocyte differentiation using the polymerase chain reaction (PCR) subtraction method [3, 4]. *Factor for adipocyte differentiation* (*Fad) 24*, *fad49*, *fad158*, *peg10* and *fad104* were shown to promote the adipocyte differentiation of 3T3-L1 cells [5–9].

The expression of *fad104*, a novel gene isolated by the PCR subtraction method, is transiently increased during adipogenesis. FAD104 has a proline-rich region, nine fibronectin type III domains and a transmembrane region. *Fad104* is known to be a member of the fibronectin type III domain containing protein (fndc) 3 family comprising *fndc3a*, *b and c. Fad104* corresponds to *fndc3b* [10]. We previously demonstrated that *fad104* positively regulated adipogenesis but negatively regulated osteoblast differentiation [11]. We also reported that *fad104*-deficient mice died at birth because of lung abnormalities [12, 13]. These results show that *fad104* is important to regulate not only adipogenesis but also lung maturation and ossification. Moreover, in addition to these findings, analyses using mouse embryonic fibroblasts (MEFs) revealed that loss of *fad104* suppressed cell adhesion, migration and proliferation [13]. These results suggest that *fad104* has essential roles in biological phenomena required for cell adhesion, migration and proliferation. Change of the potential for cell adhesion and migration is important for the invasion and metastasis of cancer cells. These findings have raised the question of whether *fad104* regulates the invasion and metastasis of cancer cells and prompted us to investigate its role in regulating malignant phenotypes of cancer cells.

In this study, we characterized the function of *fad104* in the invasion and metastasis of melanoma cells. The expression of *fad104* in highly metastatic melanoma cells was lower than that in poorly metastatic cells. In addition, *fad104* negatively regulated the invasion and metastasis of melanoma cells. Furthermore, FAD104 interacted with signal transducer and activator of transcription 3 (STAT3) and inhibited STAT3 activity. These findings indicate that *fad104* suppresses STAT3 signaling and controls the invasion and metastasis of melanoma cells.

## Materials and Methods

### Cell culture and transfection

The A375SM and A375C6 melanoma cell lines were kindly provided by Dr. Saotomo Ito and Dr. Hidetoshi Hayashi (Nagoya City University, Aichi, Japan) [14]. These cells were cultured in RPMI1640 (Wako) with 5% fetal bovine serum (FBS). A2058 melanoma cells were purchased from JCRB Cell Bank and cultured in DMEM with 10% FBS. B16F10 melanoma cells were purchased from RIKEN Cell Bank and cultured in RPMI1640 (SIGMA) supplemented with 10% FBS. MDA-MB-231 cells were kindly provided by Dr. Saotomo Ito and Dr. Hidetoshi Hayashi. The cells were cultured in DMEM with 10% FBS. All cells were maintained at 37°C with 5% CO_2_. Transfections were performed by using Lipofectamine2000 (Life Technology) according to the manufacturer’s recommendations. For serum stimulation, A375SM cells were starved for 6 hours and treated with medium containing 5% FBS.

### Generation of stable cell lines

B16F10 cells stably expressing FAD104 were generated by transfection with pCMV-3xflag-*fad104*, a FLAG-tagged FAD104 expression plasmid, and selection for neomycin resistance. B16F10 cells transfected with empty vector were used as a control.

### Adenoviral infection

Adenoviral infection has been described previously [15]. In brief, A375SM cells were infected with recombinant adenoviruses expressing FAD104 or LacZ by incubation with adenoviruses at a multiplicity of infection of 200.

### siRNA transfection

Two different human *fad104* siRNAs were purchased from Nippon EGT (si*fad104*-A and si*fad104*-B). The sequences of si*fad104*-A and si*fad104*-B were 5′-GCAGGUUAUUCUCGUUCAA-3′ and 5′-GAAGGGCCCUUCUCAGAAA-3′, respectively. Luciferase siRNA, 5′-CGUACGCGGAAUACUUCGATT-3′, was used as a control. A375SM and A375C6 cells were grown in 24-well dishes. For the transfection of si*fad104*-A, either control siRNA (20 pmol) or si*fad104*-A (20 pmol) was introduced into the cells using Lipofectamine2000 (Life Technology) in accordance with the manufacturer’s recommendations. For the transfection of si*fad104*-B, either control siRNA (40 pmol) or si*fad104*-B (40 pmol) was introduced into the cells.

### Real-time quantitative PCR (qRT-PCR)

An ABI PRISM 7000 sequence detection system (Applied Biosystems) was used for qRT-PCR. Pre-designed primers and probe sets for *fad104*, *mmp2* and *18S* rRNA were obtained from Applied Biosystems. The reaction mixture was prepared using a TaqMan Universal PCR.

### Migration and invasion assays

Migration assay was performed using BioCoat Chambers (Becton Dickinson). Transwell plates (24-well) containing 8-μm pores were coated with fibronectin. RPMI1640 containing 5% FBS was placed in the lower chamber as a chemoattractant. The cells in 250 μl of serum-free medium were plated in the upper chamber and incubated at 37°C for 24 hours. Then, the cells on the upper surface of the membrane were removed by scrubbing with cotton swabs. Chambers were fixed in 4% paraformaldehyde for 10 min and stained with crystal violet. The cells that penetrated the filter were observed with a microscope and cells from 5 randomly fields were counted. Invasion assay was carried out under the same conditions as the migration assay, except that the chambers were coated with Matrigel.

### *In vivo* metastasis assay

*In vivo* experiments were performed in accordance with the guiding principles for the care and use of laboratory animals and were approved by the ethics committee of Nagoya City University. For tail-vein metastasis assay, B16F10 cells stably expressing *fad104* (1 x 10^6^ cells/0.2 ml of PBS (-)) were injected into the tail veins of C57BL/6 mice (6 weeks old). Fifteen days later, the mice were killed by cervical spine fracture dislocation and analyzed for lung colonization. The lungs were rinsed with PBS (-) and then placed in Bouin’s solution. After 24 hours, the lungs were rinsed in PBS (-) to remove excess Bouin’s solution, and the metastatic nodules on their surface were observed.

### Western blotting

Cells were washed with PBS (-) and lysed in radio-immunoprecipitation assay (RIPA) buffer (150 mM NaCl, 50 mM Tris-HCl (pH8.0), 1% Nonidet-P40 1% sodium dodecyl sulfate (SDS), 0.5% deoxycholate) supplemented with a protease inhibitor cocktail and phosphatase inhibitor cocktail (Nacalai Tesque). After centrifugation at 15,000 rpm for 30 min, the supernatant was harvested. Equal amounts of protein were resolved using SDS-polyacrylamide gel electrophoresis (SDS/PAGE). The resolved proteins were transferred to a polyvinylidenedifluoride membrane, and probed using primary antibody and subsequently secondary antibody conjugated with horseradish peroxidase (1:10,000; Jackson ImmunoResearch Laboratories, Inc.). Specific proteins were detected using an enhanced chemiluminescence system (GE Healthcare). Primary antibodies recognizing FAD104 (1:400), phospho-STAT3 (Y705) (1:1,000; Cell Signaling), STAT3 (1:1,000; Cell Signaling) and β-actin (1:100,000; SIGMA) were used. A polyclonal FAD104 antibody was prepared in our laboratory [12]. Quantification of the band intensity of the blots was performed using NIH-Image software.

### Immunoprecipitation

A375SM cells were plated and lysed with Nonidet-P40 lysis buffer (150 mM NaCl, 50 mM Tris-HCl (pH8.0), 0.5% Nonidet-P40). Lysates were incubated with 2 μg of anti-STAT3 antibody (Santa Cruz) overnight at 4°C, and 30 μl of protein A-coupled Sepharose beads were added for binding for 4 hours at 4°C. Normal rabbit IgG was used as a negative control. The bound proteins were detected by Western blotting using anti-FAD104 antibody.

### GST pull-down assay

The pGEX-4T2 or pGEX-4T2-fad104N plasmid was transformed into BL21 *E. coli* cells. Cultures (100 ml) were grown an A_λ600 nm_ = 1.0, and protein expression was induced with 100 μM IPTG for 90 min at 30°C. Then, the suspension of BL21 cells was centrifuged at 6,000 x g for 10 min at 4°C and the pellets were resuspended in 8 ml of PBS-G (137 mM NaCl, 2.68 mM KCl, 8.10 mM NaH_2_PO_4_, 1.47 mM K_2_HPO_4_, 10% glycerol). Cells were lysed by sonication on ice. Soluble extracts were collected by centrifugation at 10,000 x g for 10 min at 4°C. GST or GST-FAD104N lysate was bound to GST-Sepharose beads overnight at 4°C. The beads were then washed three times with Nonidet P-40 lysis buffer at 4°C. Equal amounts of A375SM cell lysates were added to GST- or GST-FAD104N bound beads and subsequently rotated overnight at 4°C. After washing, the bound proteins were detected by Western blotting.

### Immunofluorescence

A375SM cells were plated onto cell disk (SUMITOMO BAKELITE) 1 day before transfection. The cells were transfected with FLAG-tagged STAT3 expression plasmid and Myc-tagged FAD104 expression plasmid using Polyethylenimine (PEI). The cell disk was fixed and incubated with mouse monoclonal anti-FLAG antibody (Sigma) for 1 hour at room temperature. After washing three times, TRITC- conjugated goat anti-mouse IgG (Sigma) and fluorescein isothiocyanate (FITC)-conjugated anti-Myc antibody (Sigma) were incubated for 1 hour at room temperature. The signals for FITC and TRITC were detected by confocal microscopy (LSM510META, Carl Zeiss).

### Luciferase reporter assay

p4xM67-tk-Luc reporter plasmid was purchased from Addgene [16]. A375SM cells were seeded in 24 well plates 24 hours before transfection. The following day, 25 ng of p4xM67-tk-Luc reporter plasmid with 75 ng of Myc-FAD104 expression plasmid or Myc-empty vector were co-transfected using PEI. To normalize the transfection efficiency, 6.25 ng of pCMV-βgal plasmid was added as an internal control. Sixteen hours after transfection, the cells were starved for 4 hours and then incubated in the presence or absence of 5% FBS or 50 ng/ml recombinant human IL-6 (Wako) for 4 hours. The cell lysates were prepared and subjected to a luciferase assay. Luciferase activity was normalized to the β-gal activity.

### Statistical tests

Analyses were performed using Excel 2010 (Microsoft Corp.) and R (http://cran.r-project.org/). The statistical significance of differences between two groups was evaluated using two-tailed Student’s t test. For multi-group analyses, significance was assessed using one-way ANOVA with *posthoc*Tukey-Kramer HSD test.

## Results

### The expression level of *fad104* in highly metastatic A375SM cells is lower than that in poorly metastatic A375C6 cells

**Fig 1:**
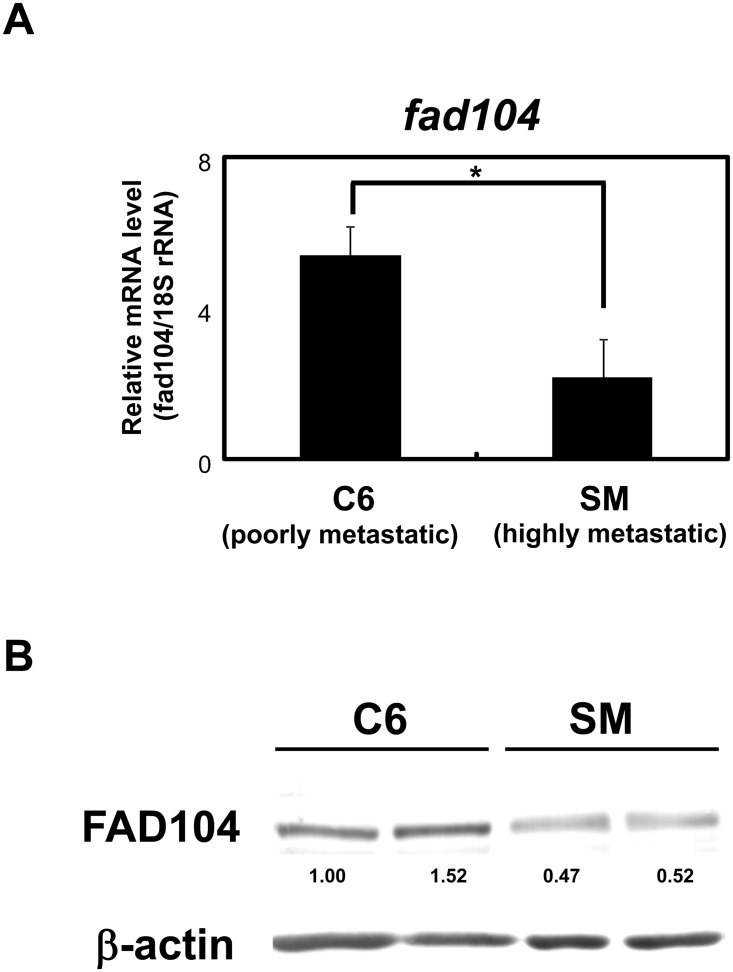
The expression level of *fad104* in highly metastatic A375SM cells is lower than that in poorly metastatic A375C6 cells. A, The mRNA expression of *fad104* in poorly metastatic A375C6 cells and highly metastatic A375SM cells. *Fad104* mRNA expression in the two cells was examined by qRT-PCR and normalized with 18S rRNA expression. Each column represents the mean with standard deviation (error bars) (n = 3). *p < 0.05. B, The protein expression of FAD104 in A375C6 cells and A375SM cells. FAD104 protein expression was examined by Western blotting. 7.5 μg of protein was loaded per lane. β-actin expression was used as a control. The ratio of protein level of FAD104/β-actin, as determined by NIH-Image software, is shown under each lane. Western blot shows the representative results in at least two independent experiments. Original uncropped images of blots are shown in Figure A in S1 File.

To elucidate the role of *fad104* in the invasion and metastasis of cancer cells, we made use of poorly metastatic A375C6 and highly metastatic A375SM variants of the A375 human melanoma cell line. We first examined the expression level of *fad104* in A375SM and A375C6 cells. Quantitative analysis of mRNA levels by qRT-PCR showed that A375SM cells expressed a lower level of *fad104* than A375C6 cells (Fig. 1A). Consistent with the lower expression of mRNA encoding *fad104*, the expression of FAD104 protein was also lower in A375SM cells (Fig. 1B). These results suggest that the expression level of *fad104* in highly metastatic A375SM cells is lower than that in poorly metastatic A375C6 cells.

### Reduction of *fad104* facilitates migration and invasion of A375C6 cells

**Fig 2:**
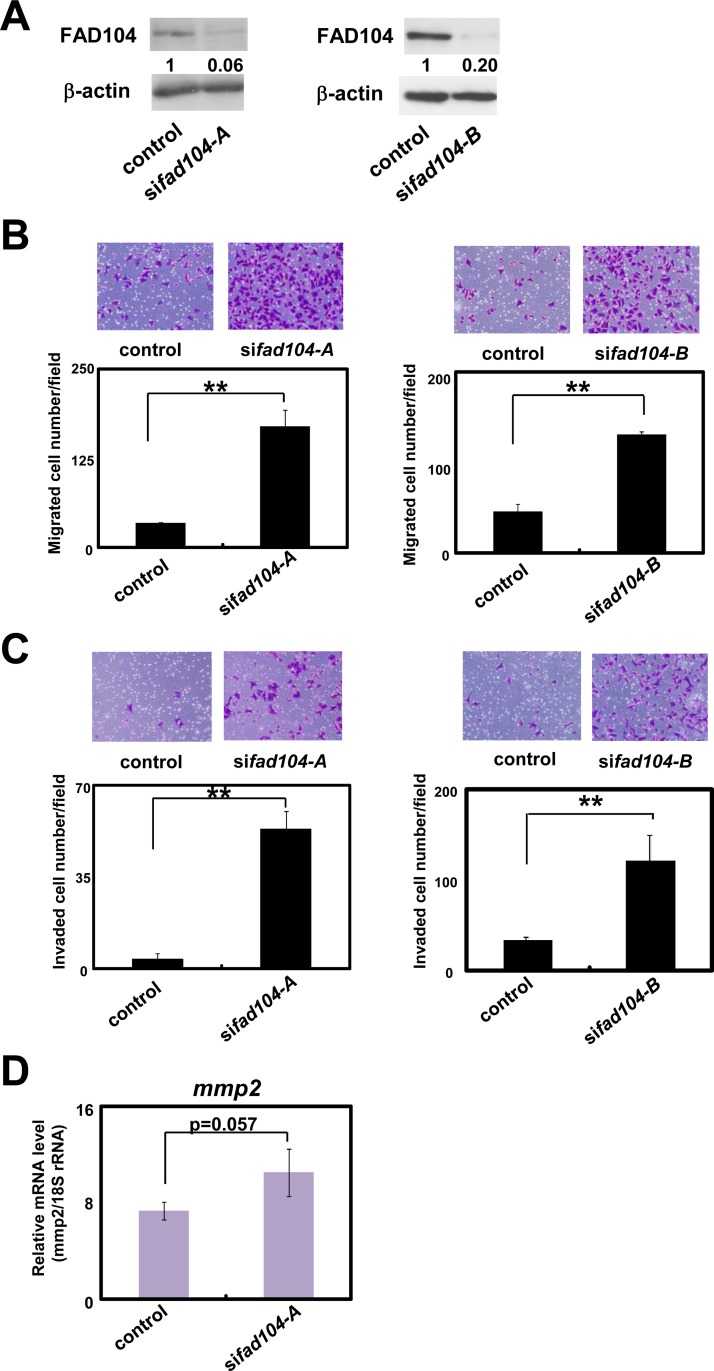
Reduction of *fad104* facilitates migration and invasion of A375C6 cells. A, Knockdown efficiency of *fad104* in A375C6 cells. A375C6 cells were transfected with two different siRNAs targeting *fad104* (si*fad104*-A, si*fad104*-B). Luciferase siRNA was used as a control. FAD104 protein expression was examined by Western blotting. 7.5 μg of protein was loaded per lane. β-actin expression was used as a control. The ratio of protein level of FAD104/β-actin, as determined by NIH-Image software, is shown under each lane. B, Transwell migration assay of *fad104* knockdown A375C6 cells and control cells. A total of 2.5x10^4^ cells were plated in the upper chamber of the filters that had been coated with fibronectin. Cells that migrated to the underside of the transwell insert were measured after 24 hours. Representative images of migrated cells are shown (upper panel). The mean number of migrated cells in the field was calculated (lower panel). C, Transwell invasion assay of A375C6 cells transfected with *fad104*siRNAs. A total of 1x10^5^ cells were plated in the upper chamber of the filters that had been coated with Matrigel. The cells that invaded the underside of the transwell insert were measured after 24 hours. Representative images of invaded cells are shown (upper panel). The mean number of invaded cells in the field was calculated (lower panel). D, The expression levels of *mmp2* mRNA in A375C6 cells transfected with si*fad104*-A were determined by qRT-PCR and normalized with 18S rRNA expression. Each column represents the mean with standard deviation (error bars) (n = 3). **p<0.01. Similar results were obtained in at least two independent experiments. Western blot shows the representative results in at least two independent experiments. Original uncropped images of blots are shown in Figure B in S1 File.

To clarify the role of *fad104* in the migration and invasion of melanoma cells, we initially transfected A375C6 cells with siRNAs targeting *fad104* (si*fad104*-A or si*fad104*-B). Both si*fad104*-A and si*fad104*-B partially suppressed the expression of endogenous FAD104 (Fig. 2A). We examined whether *fad104* regulated the migration of A375C6 cells by transwell assay. *Fad104* knockdown cells exhibited dramatically increased migration ability (Fig. 2B). Using a transwell chamber precoated with Matrigel, we next evaluated the effect of *fad104* knockdown on the invasion of A375C6 cells. As a result, knockdown of *fad104* significantly enhanced their invasion (Fig. 2C). Matrix metalloproteinases (*mmps*) are essential for tumor invasion [17]. In particular, *mmp2* is crucial for melanoma invasion and its expression level correlates directly with the pathogenesis of melanoma [18]. Therefore, we first performed qRT-PCR analysis to investigate the expression of *mmp2* in A375C6 and A375SM cells. The expression level of *mmp2* in A375SM cells was higher than that in A375C6 cells (Figure I in S1 File). We next verified the expression level of *mmp2* in *fad104* knockdown A375C6 cells. As shown in Fig. 2D, the expression level of *mmp2* was slightly increased by *fad104* knockdown. These results suggest that reduction of *fad104* facilitates the migration and invasion of A375C6 cells.

### Over-expression of FAD104 inhibits migration and invasion of A375SM cells

**Fig 3:**
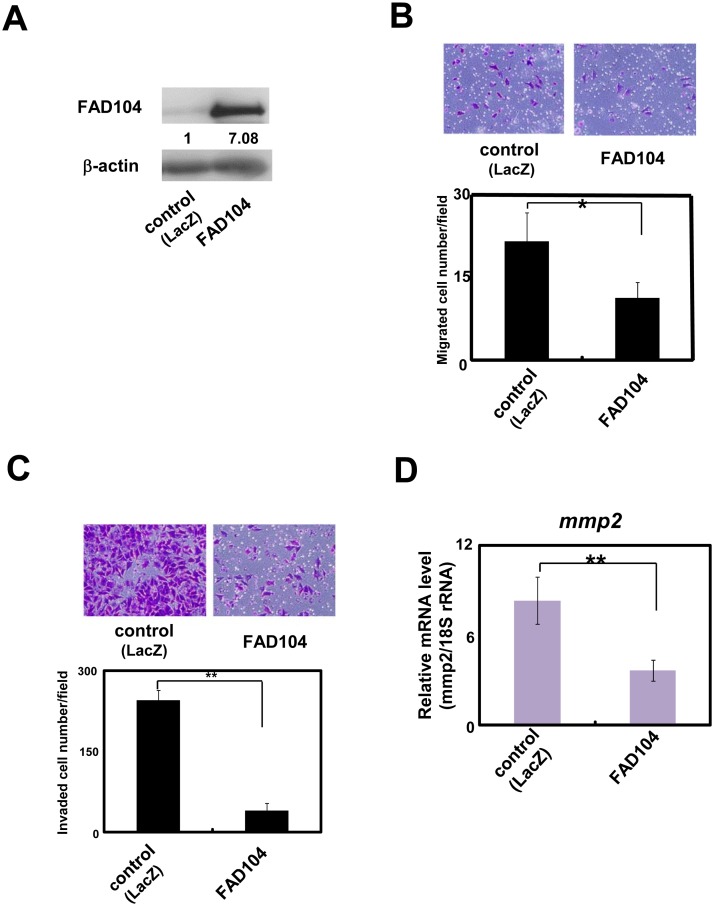
Over-expression of FAD104 inhibits migration and invasion of A375SM cells. A, FAD104 over-expression in A375SM cells. A375SM cells were infected with FAD104 or LacZ (MOI = 200). FAD104 protein expression was determined by Western blotting. 7.5 μg of protein was loaded per lane. β-actin expression was used as a control. The ratio of protein level of FAD104/β-actin, as determined by NIH-Image software, is shown under each lane. B, Transwell migration assay of FAD104 over-expressing A375SM cells and control cells. A total of 2.5x10^4^ cells were plated in the upper chamber of the filters that had been coated with fibronectin. Cells that migrated to the underside of the transwell insert were measured after 24 hours. Representative images of migrated cells are shown (upper panel). The mean number of migrated cells in the field was calculated (lower panel). C, Transwell invasion assay of A375SM cells over-expressing FAD104. A total of 1x10^5^ cells were plated in the upper chamber of the filters that had been coated with Matrigel. The cells that invaded the underside of the transwell insert were measured after 36 hours. Representative images of invaded cells are shown (upper panel). The mean number of invaded cells in the field was calculated (lower panel). D, The expression levels of *mmp2* mRNA in A375SM cells over-expressing FAD104 were determined by qRT-PCR and normalized with 18S rRNA expression. Each column represents the mean with standard deviation (error bars) (n = 3). *p < 0.05, **p <0.01. Similar results were obtained in at least two independent experiments. Western blot shows the representative results in at least two independent experiments. Original uncropped images of blots are shown in Figure C in S1 File.

Next, we assessed the effect of over-expression of FAD104 on migration and invasion. We infected A375SM cells with adenovirus encoding either FAD104 or LacZ. Exogenous expression of FAD104 in A375SM cells was attained by adenoviral transduction of the *fad104* gene (Fig. 3A). Over-expression of FAD104 significantly attenuated the number of migrated cells (Fig. 3B). In addition, FAD104-over-expressing cells also exhibited decreased invasiveness (Fig. 3C). Moreover, adenovirus-mediated FAD104 expression decreased the expression level of *mmp2* (Fig. 3D). These results suggest that over-expression of FAD104 inhibits the migration and invasion of A375SM cells.

### Reduction of *fad104* promotes invasion of A2058 cells

**Fig 4:**
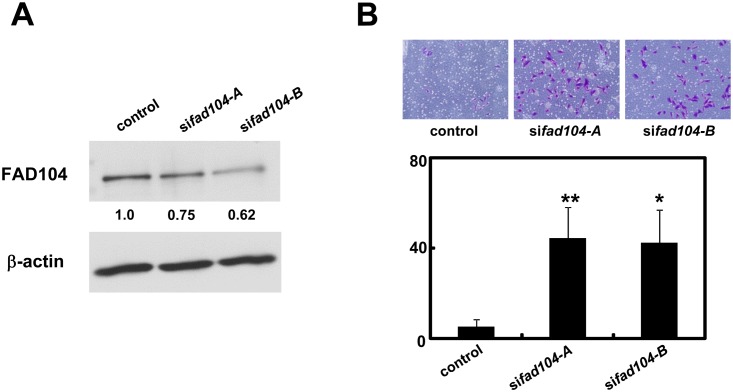
Reduction of *fad104* promotes invasion of A2058 cells. A, Knockdown efficiency of *fad104* in A2058 cells. A2058 cells were transfected with two different siRNAs targeting *fad104* (si*fad104*-A, si*fad104*-B). Luciferase siRNA was used as a control. FAD104 protein expression was examined by Western blotting. 5 μg of protein was loaded per lane. β-actin expression was used as a control. The ratio of protein level of FAD104/β-actin, as determined by NIH-Image software, is shown under each lane. B, Transwell invasion assay of A2058 cells transfected with *fad104*siRNAs. A total of 2.5x10^4^ cells were plated in the upper chamber of the filters that had been coated with Matrigel. The cells that invaded the underside of the transwell insert were measured after 24 hours. Representative images of invaded cells are shown (upper panel). The mean number of invaded cells in the field was calculated (lower panel). Each column represents the mean with standard deviation (error bars) (n = 3). *p < 0.05, **p <0.01. Similar results were obtained in at least two independent experiments. Western blot shows the representative results in at least two independent experiments. Original uncropped images of blots are shown in Figure D in S1 File.

To reveal whether *fad104* is negatively correlated with tumor progression in other melanoma cell lines, we investigated the role of *fad104* on invasion of A2058 melanoma cells, which have a highly metastatic potential, by transfecting siRNAs targeting *fad104*. Both si*fad104*-A and si*fad104*-B partially suppressed the expression of endogenous FAD104 (Fig. 4A). Using a transwell chamber precoated with Matrigel, we next evaluated the effect of *fad104* knockdown on the invasion of A2058 cells. Fig. 4B showed that knockdown of *fad104* significantly enhanced their invasion, strongly indicating that *fad104* is negatively correlated with tumor progression in a number of melanoma cell lines.

### Over-expression of FAD104 negatively regulates migration and invasion of MDA-MB-231 cells

**Fig 5:**
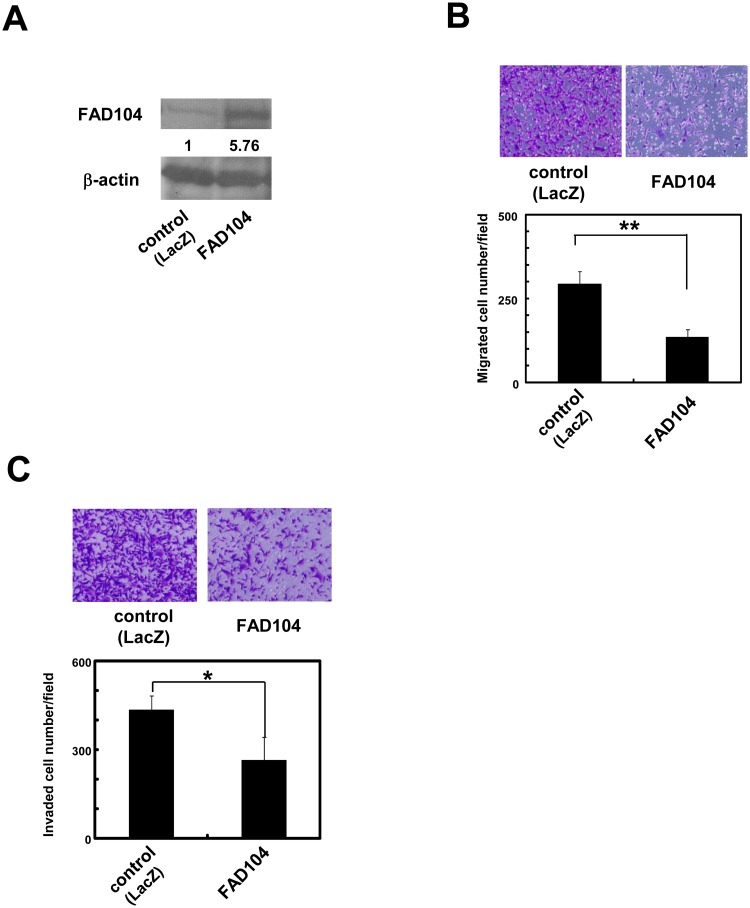
Over-expression of FAD104 negatively regulates migration and invasion of MDA-MB-231 cells. A, FAD104 over-expression in MDA-MB231 cells. MDA-MB231 cells were infected with FAD104 or LacZ (MOI = 200). FAD104 protein expression was determined by Western blotting. 7.5 μg of protein was loaded per lane. β-actin expression was used as a control. The ratio of protein level of FAD104/β-actin, as determined by NIH-Image software, is shown under each lane. B, Transwell migration assay of FAD104 over-expressing MDA-MB-231 cells and control cells. A total of 5.0x10^4^ cells were plated in the upper chamber of the filters that had been coated with fibronectin. Cells that migrated to the underside of the transwell insert were measured after 24 hours. Representative images of migrated cells are shown (upper panel). The mean number of migrated cells in the field was calculated (lower panel). C, Transwell invasion assay of MDA-MB-231 cells over-expressing FAD104. A total of 1x10^5^ cells were plated in the upper chamber of the filters that had been coated with Matrigel. The cells that invaded the underside of the transwell insert were measured after 24 hours. Representative images of invaded cells are shown (upper panel). The mean number of invaded cells in the field was calculated (lower panel). *p < 0.05, **p <0.01. Similar results were obtained in at least two independent experiments. Western blot shows the representative results in at least two independent experiments. Original uncropped images of blots are shown in Figure E in S1 File.

To assess whether *fad104* regulates migration and invasion of other cancer cells as well as melanoma cells, we next examined the effect of over-expression of FAD104 on migration and invasion of MDA-MB-231 cells derived from human breast cancer. Exogenous expression of FAD104 in MDA-MB-231 cells was attained by adenoviral transduction of the *fad104* gene (Fig. 5A). FAD104-over-expressing cells exhibited significantly decreased migration ability (Fig. 5B). In addition, over-expression of FAD104 significantly attenuated the number of invaded cells (Fig. 5C). These results suggest that over-expression of FAD104 negatively regulates migration and invasion of MDA-MB-231 cells as well as melanoma cells.

### Over-expression of FAD104 suppresses metastasis of B16F10 cells

**Fig 6:**
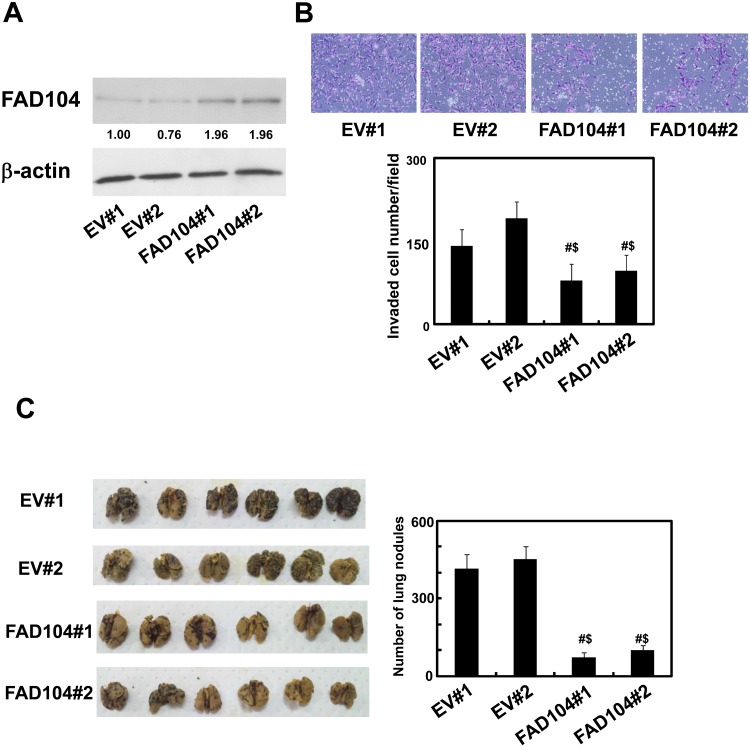
Over-expression of FAD104 suppresses metastasis of B16F10 cells. A, The protein expression of FAD104 in B16F10 cells stably expressing FAD104. FAD104 protein expression levels in FAD104-over-expressing cells (FAD104#1, #2) and control cells (EV#1, #2) were assessed by Western blotting. 5 μg of protein was loaded per lane. β-actin expression was used as a control. The ratio of protein level of FAD104/β-actin, as determined by NIH-Image software, is shown under each lane. B, Transwell invasion assay of B16F10 cells over-expressing FAD104 and control cells. A total of 2x10^5^ cells were plated in the upper chamber of the filters that had been coated with Matrigel. The cells that penetrated the filters of the chamber were measured after 24 hours. Representative images of invaded cells are shown (upper panel). The mean number of invaded cells in the field was calculated (lower panel). C, Appearance of murine lungs 15 days after injection of FAD104 over-expressing cells and control cells through the tail vein of 6-week-old mice (left panel). The quantification data of number of surface colonies are shown in right panel. Each column represents the mean with standard error (error bars) (n = 6). Statistical analyses were conducted using one-way ANOVA with Tukey-Kramer HSD test. # denotes differences between the EV#1 group and FAD104#1 or FAD104#2 group. $ denotes differences between the EV#2 group and FAD104#1 or FAD104#2 group. ^#, $^p <0.01. Similar results were obtained in at least two independent experiments. Western blot shows the representative results in at least two independent experiments. Original uncropped images of blots are shown in Figure F in S1 File.

To test the ability of FAD104 to promote lung colonization *in vivo*, we established clonal B16F10 cells stably expressing FAD104. The expression levels of FAD104 in FAD104- over-expressing cells (FAD104#1, #2) and control cells (EV#1, #2) were confirmed by Western blotting (Fig. 6A). Next, we analyzed their capacity to invade Matrigel. FAD104 significantly suppressed the number of invading B16F10 cells compared with control cells as well as A375SM cells infected with adenovirus expressing FAD104 (Fig. 6B). Using these cells, we next injected equal numbers of B16F10 with over-expression of FAD104 or control cells into the tail vein of mice. After 15 days, the mice were killed and the metastatic nodules on each lung were observed (Fig. 6C). The control cells formed numerous colonies in lung, whereas the cells stably expressing FAD104 exhibited significantly decreased ability to form colonies in lung. Taken together, these results showed that over-expression of FAD104 suppresses the metastasis of melanoma cells.

### FAD104 interacts with STAT3, and the N terminus of FAD104 is required for interaction with STAT3

**Fig 7:**
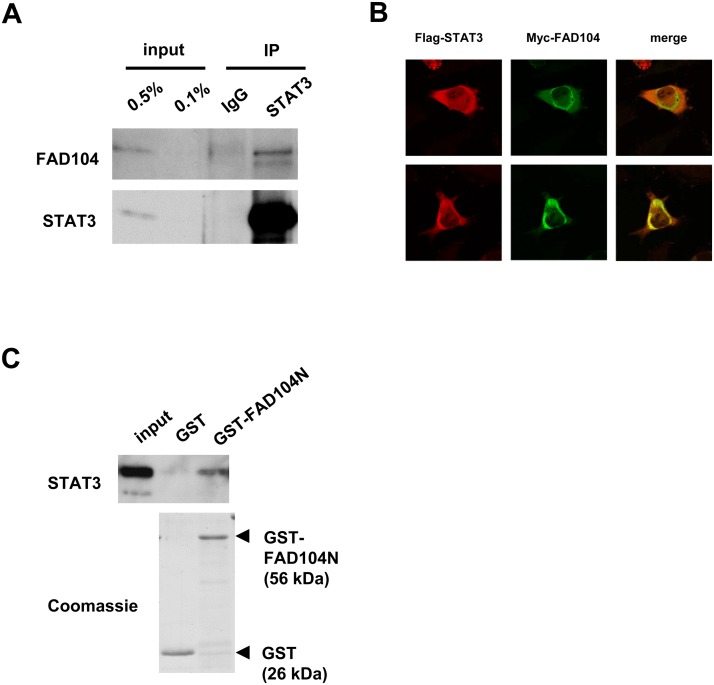
FAD104 interacts with STAT3, and the N terminus of FAD104 is required for interaction with STAT3. A, The interaction of FAD104 with STAT3 under physiological conditions. Lysates from intact A375SM cells were immunoprecipitated using antibody against STAT3. Immunoprecipitates and inputs were resolved and detected by Western blotting with anti-FAD104 antibody. B, FLAG-tagged STAT3 expression plasmid and Myc-tagged FAD104 expression plasmid were transiently introduced into A375SM cells. The signals of FLAG-STAT3 (*red*) and Myc-FAD104 (*green*) were detected with confocal microscopy. C, Interaction of the N terminus of FAD104 with STAT3 by the GST pull-down assay (upper panel). Cell lysates prepared from A375SM cells were used for a GST pull-down assay with GST or GST-FAD104N (1–277 a.a.). Coomassie Blue staining of the GST proteins (lower panel). Bound protein samples were immunoblotted with anti-STAT3 antibody. The input volume was 0.5% of that of the cell lysate for the pull-down assay. Each Western blot shows the representative results in at least two independent experiments. Original uncropped images of blots are shown in Figure G in S1 File.

We next aimed to obtain a mechanistic insight into how FAD104 regulates the invasion and metastasis of melanoma cells. FAD104 contains proline-rich and fibronectin type III domains, which are mediated by protein-protein interaction and signal transduction [19–22]. In particular, the N terminus of FAD104 has proline-rich motifs that contain several binding sites for SH3 (PXXP) and type I WW domains (PPXP and PXXY). Therefore, FAD104 might interact with a factor involved in invasion and metastasis. It is reported that STAT3 is the central signaling protein that enhances the growth and progression of melanoma cells [23–25]. Furthermore, STAT3 regulates the expression of *mmp2* in melanoma cells [26]. Moreover, Young et al. reported that the central linker region of STAT3 may potentially mimic the SH3 and/or type I WW domains [27]. Therefore, we assessed whether FAD104 interacted with STAT3 in melanoma cells under physiological conditions. Immunoprecipitation assay showed that FAD104 interacted with STAT3 in melanoma cells (Fig. 7A). Next, we examined whether FAD104 colocalized with STAT3 in melanoma cells. It is reported that STAT3 localizes in cytoplasm and to a lesser degree in the nucleus in normal condition [28]. Indeed, we observed that STAT3 localized in cytoplasm and nucleus in A375SM cells. Moreover, the cytoplasmic localization of FLAG-STAT3 was partially merged with Myc-FAD104 (Fig. 7B). Using GST pull-down assay, we next examined whether the N terminus of FAD104 that contains a proline-rich motif is required for interaction with STAT3, and found that GST-FAD104N (1–277 a.a.) bound to STAT3 (Fig. 7C). Taken together, these results suggest that FAD104 interacts with STAT3 in melanoma cells and the N terminus of FAD104 is required for this interaction.

### *Fad104* negatively regulates the phosphorylation level of STAT3 in melanoma cells

**Fig 8:**
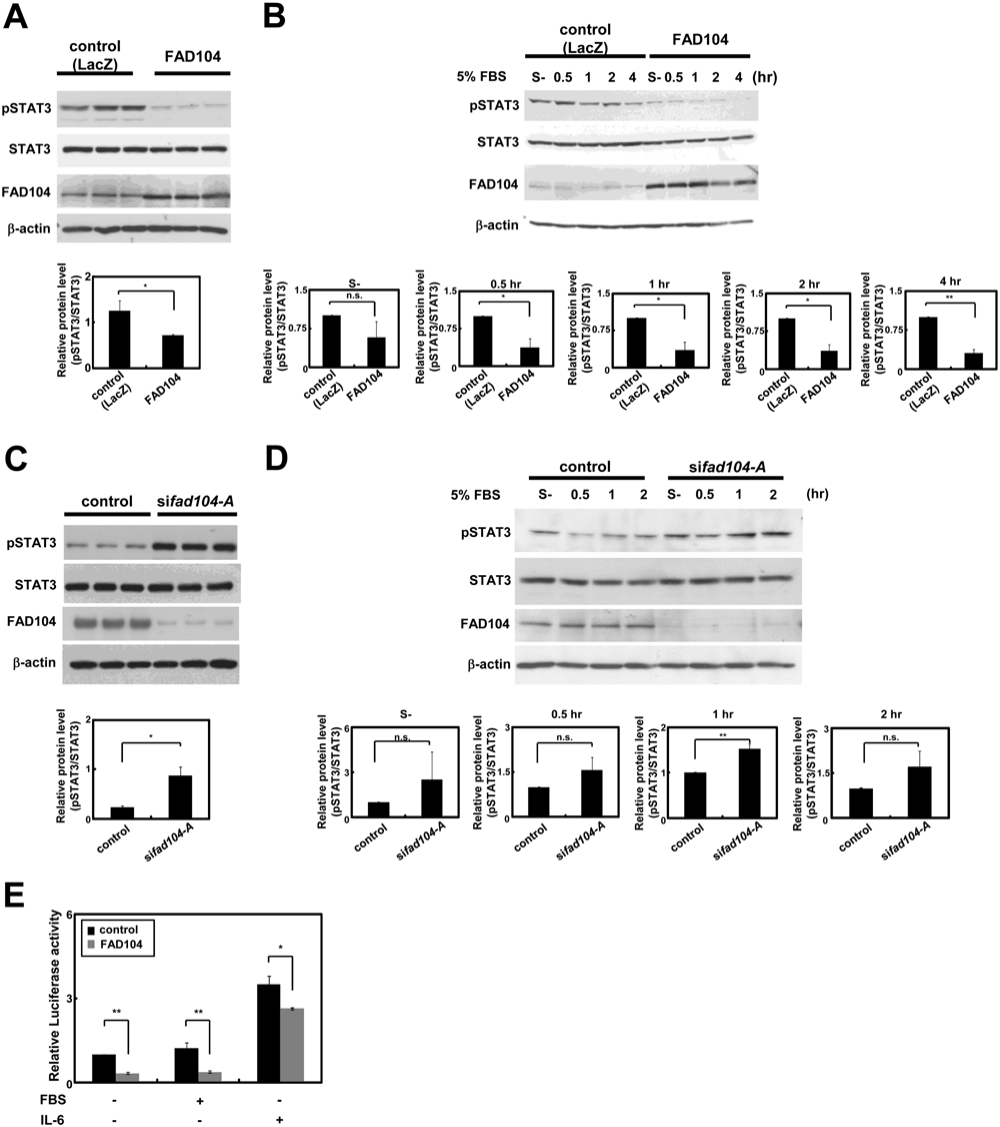
*Fad104* negatively regulates the phosphorylation level of STAT3 in melanoma cells. A, The phosphorylation level of STAT3 in A375SM cells over-expressing FAD104. The phosphorylation level of STAT3 and total protein level of STAT3 were detected by Western blotting (upper panel). 5 μg of protein was loaded per lane. β-actin expression was used as a control. Intensities of signals from phospho-STAT3 and total STAT3 were quantified by NIH-Image software. Quantification of the reduction rate of phospho-STAT3 was shown (lower panel). B, The phosphorylation level of STAT3 in A375SM cells infected with FAD104 after treatment with medium containing 5% FBS. At indicated points after serum treatment, the phosphorylation and total protein levels of STAT3 were detected by Western blotting (upper panel). 5 μg of protein was loaded per lane. β-actin expression was used as a control. Intensities of signals from phospho-STAT3 and total STAT3 were quantified by NIH-Image software. Quantifications of the reduction rate of phospho-STAT3 at each time point were shown (lower panel). C, The phosphorylation level of STAT3 in A375SM cells transfected with si*fad104*-A. The level of phosphorylation and total protein level of STAT3 were detected by Western blotting (upper panel). 5 μg of protein was loaded per lane. β-actin expression was used as a control. Intensities of signals from phospho-STAT3 and total STAT3 were quantified by NIH-Image software. Quantification of the promotion rate of phospho-STAT3 was shown (lower panel). D, The phosphorylation level of STAT3 in *fad104* knockdown A375SM cells after treatment with medium containing 5% FBS. At indicated points after serum treatment, the level of phosphorylation and total protein level of STAT3 were detected by Western blotting (upper panel). 5 μg of protein was loaded per lane. β-actin expression was used as a control. Intensities of signals from phospho-STAT3 and total STAT3 were quantified by NIH-Image software. Quantifications of the promotion rate of phospho-STAT3 at each time point were shown (lower panel). E, A375SM cells were transfected with 4xM67-tk-Luc luciferase reporter plasmid, in the presence or absence of Myc-tagged FAD104 expression plasmid. At 16 hours after transfection, the cells were starved for 4 hours and then incubated in the presence or absence of 5% FBS or IL-6 for 4 hours. The cell lysates were prepared and subjected to a luciferase assay. Luciferase activity was normalized to the β-gal activity. The relative luciferase activity was calculated from the mean value relative to control (leftmost lane), set as 1. Each column represents the mean with standard deviation (error bars) (n = 3). *p < 0.05, **p <0.01. Each Western blot shows the representative results in at least three independent experiments. Original uncropped images of blots are shown in Figure H in S1 File.

STAT proteins form homo- and hetero-dimers after the phosphorylation of tyrosine residues. Phosphorylated STAT translocates into the nucleus and regulates the expression of target genes [29]. Therefore, we tested whether *fad104* regulated the phosphorylation level of STAT3 in melanoma cells. The phosphorylation level of STAT3 significantly decreased in A375SM cells infected with adenovirus expressing FAD104, while the total level of STAT3 protein was only slightly decreased in FAD104 over-expressing cells (Fig. 8A). Furthermore, we examined whether FAD104 negatively regulated the phosphorylation level of STAT3 under serum treatment. FAD104-over-expressing A375SM cells were incubated with serum-free medium and treated with medium containing 5% FBS. As shown in Fig. 8B, the phosphorylation level of STAT3 was promoted after treatment with medium containing 5% FBS in control cells. However, the phosphorylation level of STAT3 in A375SM cells over-expressing FAD104 significantly declined compared with that of control cells.

Finally, we assessed the effect of *fad104* knockdown on the activation of STAT3. As shown in Fig. 8C, the phosphorylation level of STAT3 in *fad104* knockdown cells was significantly promoted compared with that of control cells, whereas the total level of STAT3 protein was nearly unchanged. We also investigated the STAT3 activity under serum treatment. While the total level of STAT3 protein was unchanged, the phosphorylation level of STAT3 at each time point was increased by *fad104* knockdown. In particular, phosphorylation level of STAT3 at 1 hour after treatment with 5% FBS was significantly increased by *fad104* knockdown (Fig. 8D). We next investigated the effect of over-expression of *fad104* on STAT3 transcriptional activity. We introduced a STAT3 reporter construct (4xM67-tk-Luc) into A375SM cells with Myc-tagged FAD104 expression plasmid or empty vector. As a result, transcriptional activity of STAT3 was significantly reduced by over-expression of *fad104* (Fig. 8E). Taken together, these results strongly indicated that *fad104* negatively regulates the phosphorylation level and transcriptional activity of STAT3 in melanoma cells.

## Discussion

In this report, we show that *fad104* suppressed the invasion and metastasis of melanoma cells via the inhibition of STAT3 activity. The development of metastatic disease is highly complex. Tumor cells detach from the primary tumor mass; migrate and invade through the extracellular matrix, basement membrane and endothelial wall; and circulate in the vascular system, before establishing themselves in the target organ [30]. STAT3 is constitutively activated at 50–90% frequency in diverse human cancers. In fact, the majority of melanoma cell lines and tumor specimens display constitutively activated STAT3 [31]. It is reported that the inhibition of STAT3 activity by JSI-124 (cucurbitacin I), an inhibitor of STAT3, suppressed tumor growth of B16F10 melanoma cells [32]. Moreover, dominant negative STAT3 attenuates the potency of invasion and metastasis of melanoma [23–26]. Thus, regulation of STAT3 activity may enhance the efficacy of melanoma therapy. Here, we showed that FAD104 interacted with STAT3 and suppressed the phosphorylation level of STAT3. Therefore, FAD104 may mask the STAT3 phosphorylation site. It is necessary to reveal the region of STAT3 that is important for interaction with FAD104. Moreover, activation of STAT3 is tightly controlled by various molecules, such as protein tyrosine phosphatases (e.g. PTPMeg2), inhibitory factors (e.g. protein inhibitors of activated STAT (PIAS)), and negative feedback factors (e.g. suppressor of cytokine signaling (SOCS)) [33–35]. Therefore, it is necessary to examine whether *fad104* regulates activation of STAT3 via cooperation with these molecules.

It is reported that tenascin-C is highly expressed in melanoma cells [36]. Other researchers demonstrated that RhoA and RhoC were also over-expressed in highly metastatic melanoma cells [37]. Thus, the expression of the factors involved in invasion and metastasis frequency correlates with tumor progression. Here, we indicated that the level of *fad104* is decreased in highly metastatic A375SM cells, implying that the expression of *fad104* is associated with the progression of melanoma cells. Furthermore, we previously reported that the expression of *fad104* increased in the early stage of adipogenesis and decreased during osteogenesis [9, 11]. It is possible that the change of expression level of *fad104* is important for promotion of the adipogenesis and inhibition of the osteogenesis, invasion and metastasis of cancer cells. However, the mechanisms by which the expression of *fad104* is regulated remain unclear. In melanoma cells, in addition to STAT3, various transcription factors such as the p53 family, nuclear factor kappa B (NF-κB), activating transcription factor-1/2 (ATF-1/2), cAMP-responsive element-binding (CREB) protein and activator protein-2α (AP-2α) directly control the expression of genes contributing to adhesion, migration, matrix degradation and cell survival [38–42]. Therefore, we need to perform the promoter analyses to clarify whether these factors regulate the expression of *fad104*. In addition, single-nucleotide polymorphism (SNP) analyses should be performed to investigate the role of *fad104* in metastasis.

MMP2 is a member of the matrix metalloproteinase family which consists of zinc-dependent enzymes that degrades different extracellular matrix (ECM) proteins [43]. MMP2 is often over-expressed in malignant tumors. It is reported that MMP2 was frequently associated with malignant progression and poor prognosis in melanoma [43–45]. Here, we showed that the over-expression of FAD104 decreased the expression of *mmp2. Mmp2* expression is regulated by not only STAT3 but also other transcription factors such as FoxO3a [46]. It is necessary to examine the mechanisms by which FAD104 regulates the expression of *mmp2*.

We showed that *fad104* suppressed migration and invasion of not only melanoma cells but also breast cancer cells (Fig. 5). Therefore, it is possible that *fad104* plays an important role for regulation of migration and invasion of various cancer cells. It is necessary to clarify whether *fad104* regulates migration and invasion of other cancer cells such as lung and prostate cancer.

FAD104 is also known to be FNDC3B [10]. It is reported that miRNA-143 promotes invasion and metastasis of hepatocellular carcinoma and prostate cancer cells by repression of FAD104/FNDC3B expression [47, 48]. However, how FAD104/FNDC3B regulates invasion and metastasis remains unknown. In this study, we elucidated for the first time that FAD104 regulated invasion and metastasis of melanoma cells through inhibition of STAT3 activity. It is needed to clarify the mechanism by which FAD104/FNDC3B regulates invasion and metastasis of liver and prostate cancer cells. Moreover, it remains unknown whether miR-143 regulates metastasis of melanoma cells, and FAD104/FNDC3B expression. Therefore, it is definitely required to investigate the effect of miR-143 on invasion and metastasis of melanoma and breast cancer cells in future study.

In summary, we demonstrated that *fad104* suppresses the invasion and metastasis of melanoma cells and is closely involved in negative regulation of the STAT3 signaling pathway. Further analyses of the mechanism by which *fad104* regulates invasion and metastasis should help to elucidate the whole picture of the invasion and metastasis of cancer cells.

## Supporting Information

S1 File Figures A through I. (PDF)
