# Supplementary material for: Fad104, a Positive Regulator of Adipocyte Differentiation, Suppresses Invasion and Metastasis of Melanoma Cells by Inhibition of STAT3 Activity
Source: PLoS One. 2015 Feb 11;10(2):e0117197. doi: 10.1371/journal.pone.0117197 (PMC4324941; doi:10.1371/journal.pone.0117197)
Supplement: S1 File — (PDF) [file pone.0117197.s001.pdf]

Fig. 1B: FAD104

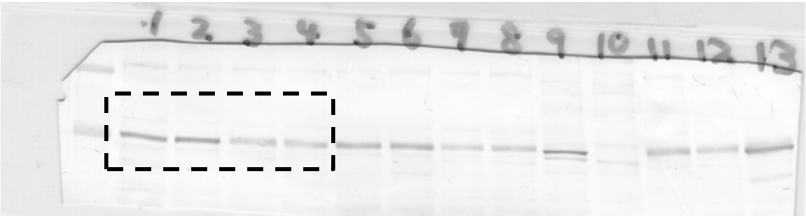

Fig. 1B: β-actin

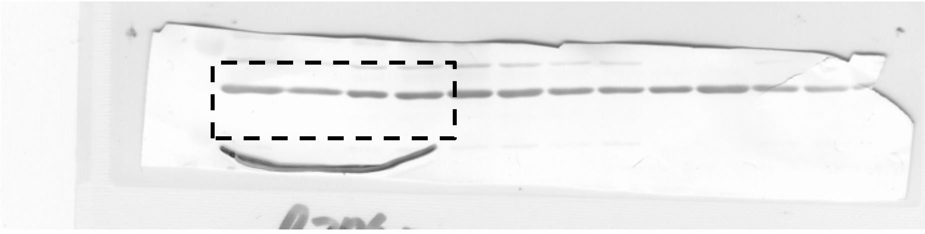

**Figure A. Original uncropped images of blots.**
Boxes highlighted lanes used in Fig. 1.

**Fig. 2A left panel: FAD104 and β-actin**

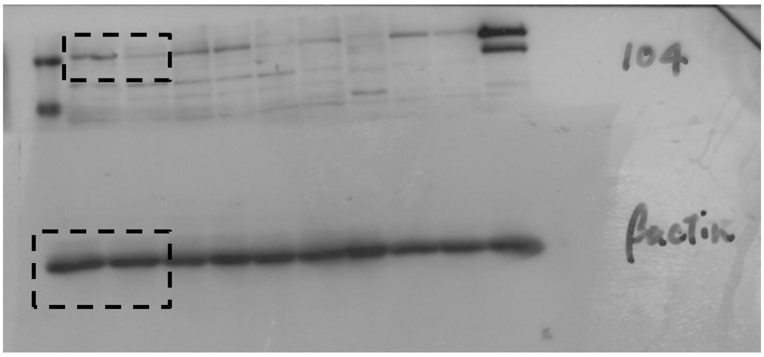


**Fig. 2A right panel: FAD104**

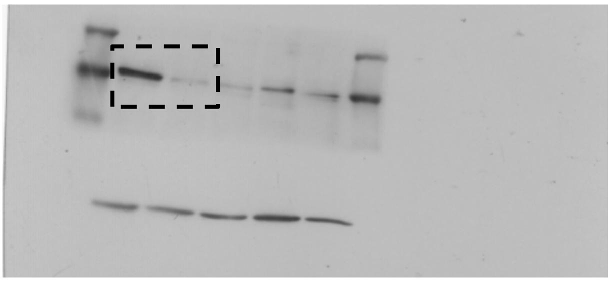

**Fig. 2A right panel: β-actin**

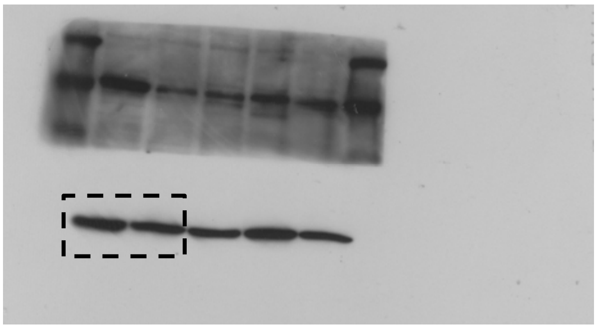

**Figure B. Original uncropped images of blots.**
Boxes highlighted lanes used in Fig. 2.

**Fig. 3A: FAD104 and β-actin**

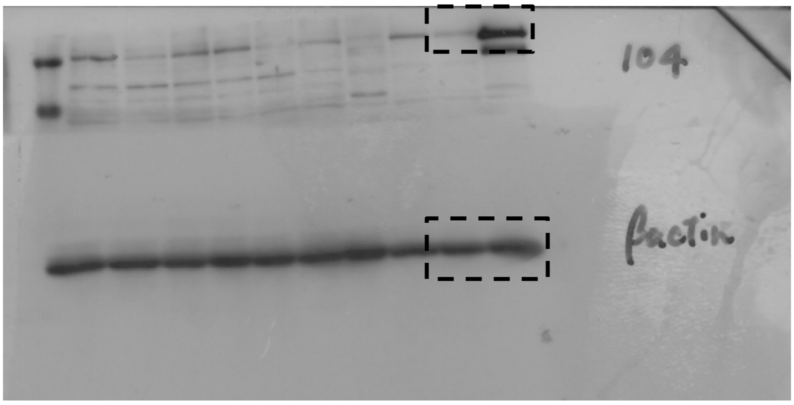


**Figure C. Original uncropped images of blots.**
Boxes highlighted lanes used in Fig. 3.

Fig. 4A: FAD104 and β-actin

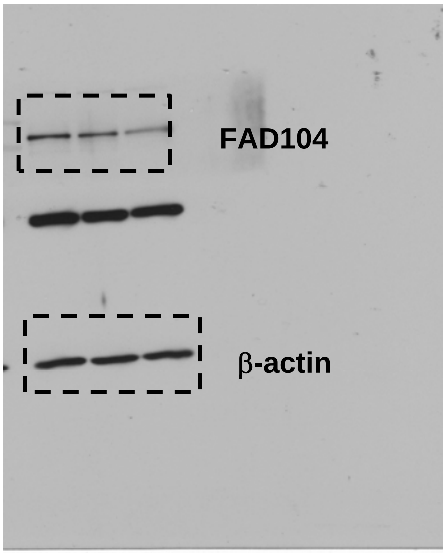


**Figure D. Original uncropped images of blots.**
Boxes highlighted lanes used in Fig. 4.

**Fig. 5A: FAD104 and β-actin**

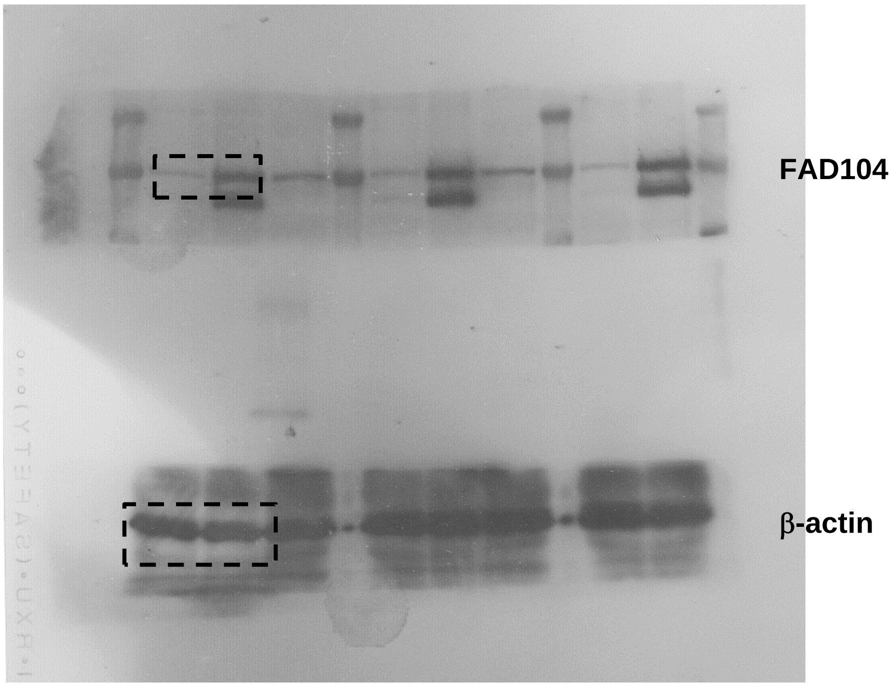


**Figure E. Original uncropped images of blots.**
Boxes highlighted lanes used in Fig. 5.

**Fig. 6A:FAD104**

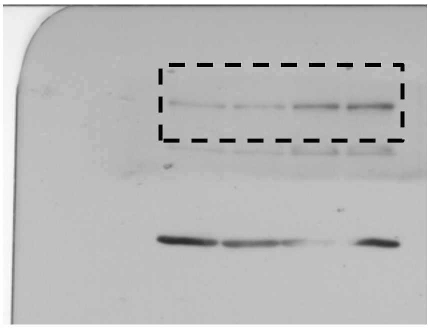

**Fig. 6A: β-actin**

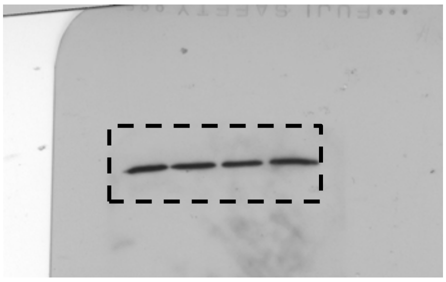

**Figure F. Original uncropped images of blots.**
Boxes highlighted lanes used in Fig. 6.

**Fig. 7A: FAD104 and STAT3**

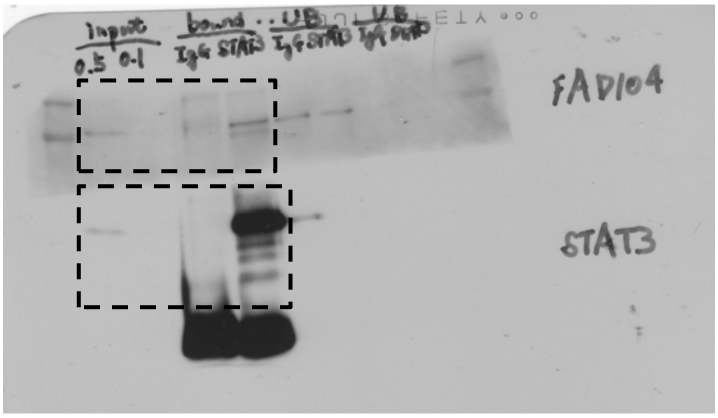


**Fig. 7C: upper panel**

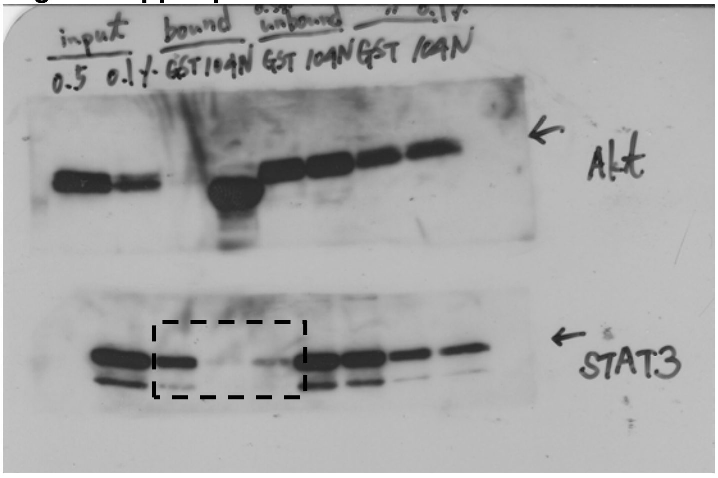


**Fig. 7C: lower panel**

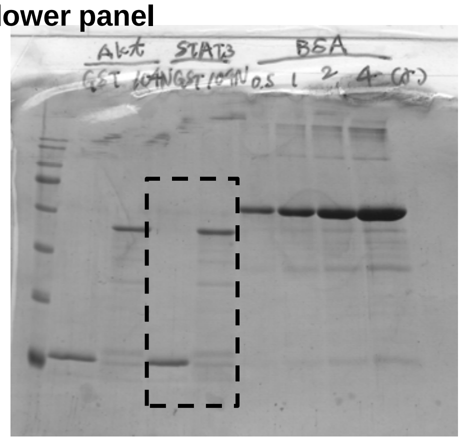


**Figure G. Original uncropped images of blots.**
Boxes highlighted lanes used in Fig. 7.

Fig. 8A: FAD104 and β-actin

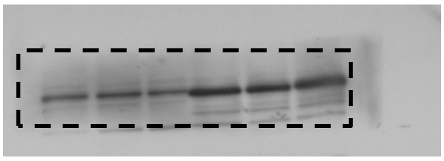

Fig. 8A: pSTAT3

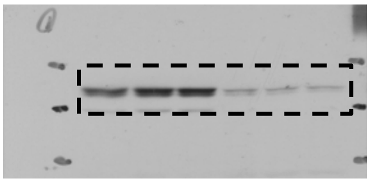

Fig. 8A: β-actin

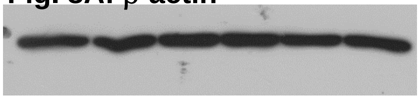

Fig. 8A: STAT3

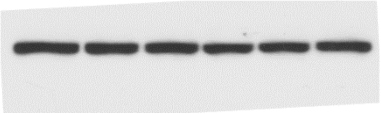

Fig. 8B: pSTAT3 and β-actin

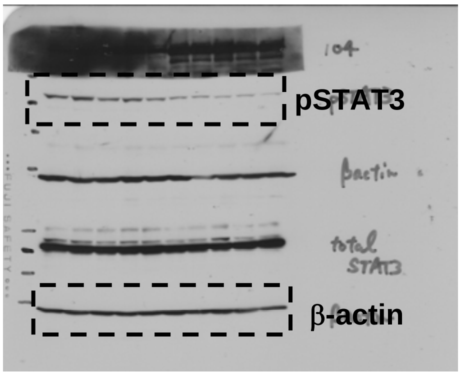


Fig. 8B: FAD104 and STAT3

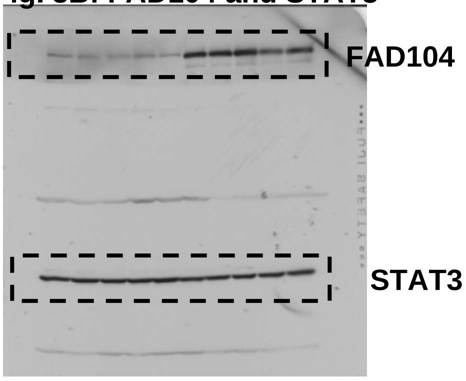


Fig. 8C: pSTAT3

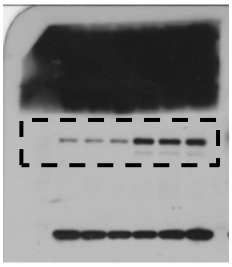

Fig. 8C: β-actin

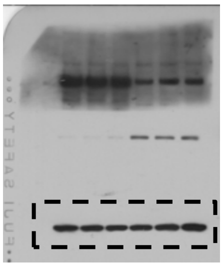

Fig. 8C: FAD104

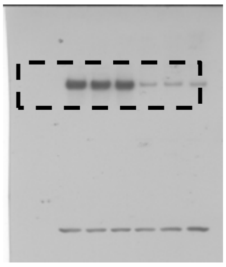

Fig. 8C: STAT3

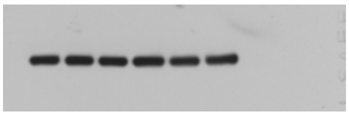

Fig. 8D: FAD104, STAT3 and β-actin

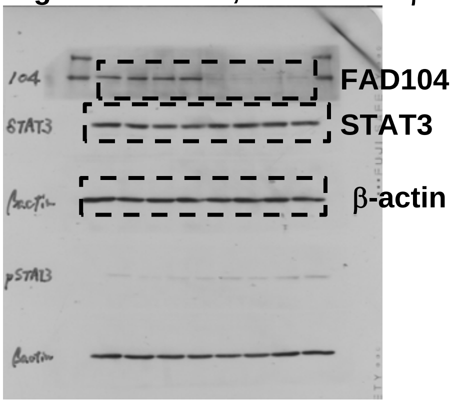


Fig. 8D: pSTAT3

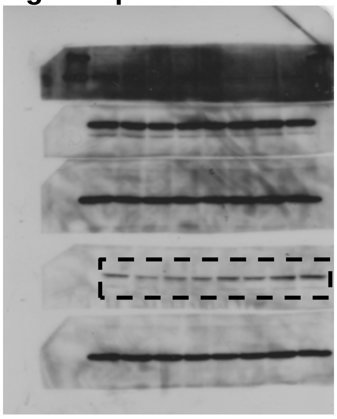

**Figure H. Original uncropped images of blots.**

Boxes highlighted lanes used in Fig. 8.

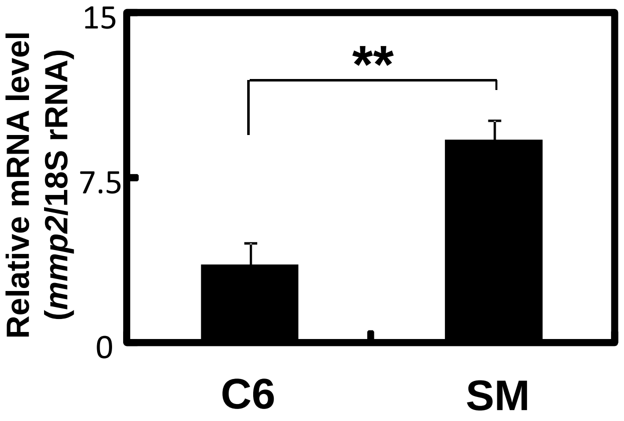


**Figure I. The expression level of mmp2 of A375SM cells was higher than that of A375C6 cells.**

The expression levels of *mmp2* mRNA between in A375SM cells and A375C6 cells were determined by qRT-PCR and normalized with 18S rRNA expression. Each column represents the mean with standard deviation (error bars) (n = 3). **p <0.01.
